# The Association of Perioperative Serum Lactate Levels with Postoperative Delirium in Elderly Trauma Patients

**DOI:** 10.1155/2019/3963780

**Published:** 2019-11-16

**Authors:** Cheol Lee, Juhwan Lee, Hyunho Cho, Jaekyeong Song, Hojung Jung, Xiao Ma, Jihyo Hwang

**Affiliations:** ^1^Department of Anesthesiology and Pain Medicine, Wonkwang University School of Medicine, Iksan, Republic of Korea; ^2^Department of Orthopaedic Surgery, Gangnam Sacred Heart Hospital, Hallym University School of Medicine, Seoul, Republic of Korea

## Abstract

**Background:**

Several studies have shown the utility of lactate level as a predictor of early outcomes in trauma patients. We conducted this study to evaluate the association of perioperative serum lactate levels with postoperative delirium (POD) in elderly trauma patients.

**Materials and Methods:**

This study included 466 elderly trauma patients with measurements of serum lactate levels on admission and 1 h after surgery. The associations of POD with serum lactate levels (on admission and 1 h after surgery) and lactate clearance were analyzed using Kendall's correlation. Perioperative serum lactate levels and lactate clearance as predictors of POD were evaluated using univariate and multivariable analyses.

**Results:**

The incidence of POD in the present study was 38.1%. Serum lactate levels on admission and at 1 h after surgery were significantly higher in major trauma than in minor trauma. In univariate analysis of perioperative serum lactate levels and lactate clearance as predictors of POD, the odds ratio (OR) for serum lactate level on admission was 4.19 (*P* < 0.01, 2.91 < 95% confidence interval (CI) < 6.02) and that 1 h after surgery was 3.83 (*P* < 0.01, 2.79 < 95% CI < 5.25); however, the OR for serum change of lactate level was 0.99 ((*P* < 0.09, 0.99 < 95% CI < 1.00). In multivariable analysis for predictors of POD, the OR for serum lactate level on admission was 2.40 (*P* < 0.09, 0.87 < 95% CI < 6.7), that for serum lactate 1 h after surgery was 2.83 (*P*=0.01, 1.28 < 95% CI < 6.24), that for ICU admission was 3.01 (*P*=0.01, 2.09 < 95% CI < 6.03), and that for ISS was 1.47 (*P* < 0.01, 1.27 < 95% CI < 3.70).

**Conclusions:**

Taking together the results of univariate and multivariable analyses, serum lactate level 1 h after surgery may be used as a prediction model of POD development in elderly trauma patients.

## 1. Introduction

Elevated lactate levels are not only observed in severely hypotensive trauma patients but also may be seen in normotensive patients with multiple injuries. Thus, serum lactate level can be used as a marker of the balance between oxygen demand and availability, and changes in serum lactate levels have been shown to be an effective prognostic biomarker of mortality in critically ill trauma patients, including those with stable vital signs [1].

Postoperative delirium (POD) is the most common neurologic complication of major surgery in patients older than 65 years. The development of delirium reflects both the severity of illness and advancing age of the patient [2, 3]. The incidence of POD after major surgery varies between 10% and 70% depending on the criteria used for diagnosis, the population studied, and the type of surgical procedure [4–6]. The underlying pathophysiology of POD is not well understood. The suggested mechanisms include neuronal aging, oxidative stress, neuroinflammation, neurotransmitter deficiency, diurnal dysregulation, neuroendocrine activation, and brain network connectivity change [7].

In trauma medicine, both patients with established delirium, wherein the delirium has preceded or sometimes led to trauma, and those with incident delirium, which develops as a consequence of trauma and hospitalization process, are encountered [8, 9]. Hypoperfusion in trauma patients remains difficult to diagnose and causes an adverse immunoinflammatory response [1]. The precise role of serum lactate level in the development of POD in trauma patients remains difficult to determine. Few studies have reported the association of serum lactate level with POD in older patients with trauma. One study on the incidence and risk factors of early delirium after cardiac surgery reported that perioperative serum lactate levels were significantly higher in patients with delirium than those in patients without delirium, as a secondary outcome [10].

We hypothesized that lactate is a marker of the balance between oxygen demand and availability leading to an immunoinflammatory response. Therefore, the elevated serum lactate levels in elderly trauma patients might be associated with the incidence of POD. The aim of our study was to assess the effect of serum lactate level on the development of POD in elderly trauma patients, as a primary outcome.

## 2. Materials and Methods

### 2.1. Study Design

In this retrospective cohort study, we evaluated the medical records of all consecutive geriatric (age ≥65 years) trauma patients admitted from January 1, 2009, through June 30, 2018, to a level 1 single trauma center that provides the highest level of care and comprehensive service for trauma patients. Ethical approval for this study (registration no. 2018-08-24) was provided by the institutional review board in September 2018.

### 2.2. Patient Selection

**Figure 1:**
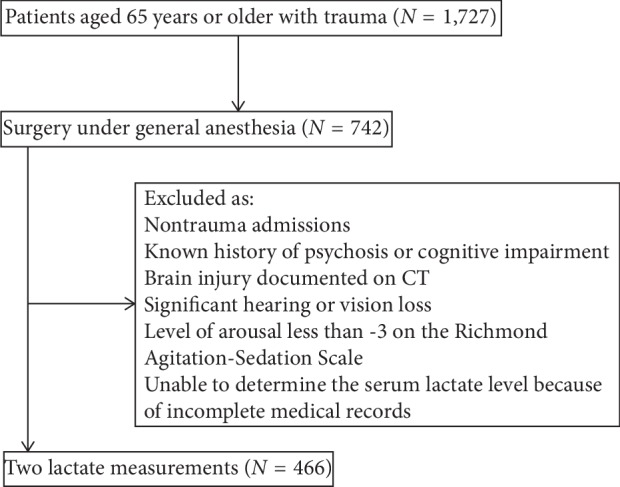
Diagram of study flow.

Patients >65 years of age who were with trauma and potential mild traumatic brain injury, classified as class I to IV according to the American Society of Anesthesiologists (ASA) classification, and scheduled for surgery under general anesthesia were considered eligible for inclusion in this study. We excluded patients who had nontrauma admissions, known history of psychosis or cognitive impairment, brain injury documented on computed tomography (CT), significant hearing or vision loss, and a level of arousal less than −3 on the Richmond Agitation-Sedation Scale. We also excluded patients for whom we were unable to determine the serum lactate level because of incomplete medical records (Figure 1). The data for the present study were collected from the medical records of eligible patients and included demographics, mechanism of injury, injury severity score (ISS), and perioperative variables (time from admission to surgery, serum lactate levels on admission and 1 h after surgery, lactate clearance, vasoactive agents used, and incidence of POD).

### 2.3. Blood Lactate Measurements

Arterial blood lactate levels were measured on admission to the emergency and trauma center and at 1 h after surgery. Change of lactate level was calculated using the following equation:

(1) $$\begin{array}{c} \text{change of lactate level }=\frac{\left[\text{lactate on hospital admission}\right]-\left[\text{lactate }1\text{ h after surgery}\right]}{\left[\text{lactate on hospital admission}\right]}\times 100\text{.} \end{array}$$

### 2.4. Assessment of POD

Postoperative patients at our hospital are assessed in every 8 hours by nurses in the general ward or intensive care unit (ICU) with the 4 “A's” test (4AT) to screen for delirium [11]. The brevity and simplicity of the 4AT have encouraged its use by nurses in routine clinical practice. After detecting delirium, the nurses report to the doctor and psychiatrists, who confirm the diagnosis of delirium with a Confusion Assessment Method (CAM) for the ICU. This well-validated tool evaluates for acute onset of changes or fluctuations in mental status, inattention, and either disorganized thinking or an altered level of consciousness. The CAM-ICU [12] has been validated specifically for use in the trauma ICU and general ward. POD was assessed as delirium observed from the day just after the operation to the day before discharge.

### 2.5. Statistical Analysis

Statistical analyses were performed using SPSS version 18.0 (SPSS Inc., Chicago, IL, USA). Variables in the 2 groups were analyzed using Pearson chi-square tests and Students' *t*-test. Correlation between variables was analyzed using Kendall's tau-b. To estimate the independent relationship of perioperative serum lactate levels and change of lactate level to POD, we performed univariate and multivariable logistic regression. We assessed predictor variables to ensure that they met the linearity assumption of logistic regression, those that did not were categorized. For model selection, we identified candidate predictors with a univariate *P* < 0.05 and used a stepwise forward selection procedure. We then assessed models for problems with collinearity and examined key variables for effect modification using interaction terms. Values are expressed as mean ± standard deviation or number (%) of patients. Odds ratios (ORs) with 95% confidence intervals (CIs) were obtained. For all analyses, statistical significance was defined as *P* < 0.05.

## 3. Results

**Figure 2:**
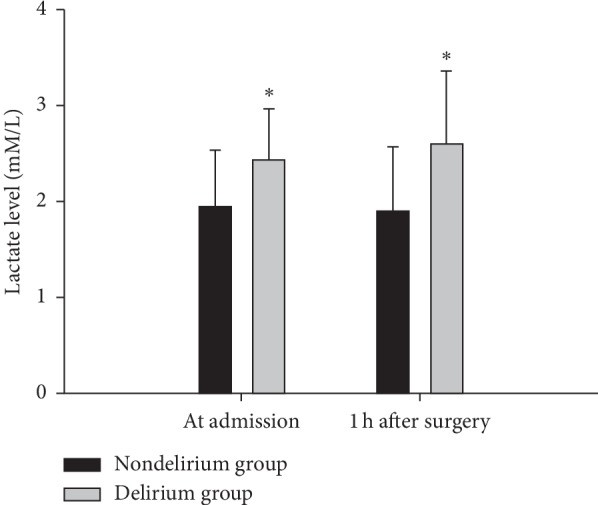
Perioperative serum lactate levels between nondelirium group and delirium group.

Of 1,727 elderly trauma patients admitted during the study period, 742 patients underwent surgery. A total of 466 patients had 2 lactate measurements (on admission and 1 h after surgery) and thus had a calculable change of lactate level. Therefore, 446 patients were finally included in this study (Figure 1). Of them, 170 (38.1%) patients developed POD. Age, sex, weight, Glasgow Coma Scale score, and change of lactate level were not significantly different between patients with and without POD. The incidences of alcohol abuse history, delirium history, hypertension history, and polypharmacy were significantly higher in patients with POD than those in patients without POD. There were significant differences in the mechanism of injury, duration of surgery, duration of anesthesia, patient-controlled analgesia volume, time from admission to surgery, intraoperative transfusion and fluid administered, hemoglobin level 1 h after surgery, serum lactate level on admission and 1 h after surgery (Figure 2), vasoactive agents used, and ICU admission between patients with and without delirium (Table 1).

Correlations between postoperative delirium and serum lactate level on admission (*r* = 0.31, *P* < 0.01) and serum lactate level 1 hour postoperatively (*r* = 0.36, *P* < 0.01) were moderate positive (Table 2). The incidence of POD in major trauma was significantly higher than that in minor trauma. Serum lactate level on admission and serum lactate level 1 hour after surgery in delirium with major trauma were significantly higher than those in delirium with minor trauma (Table 3).

**Figure 3:**
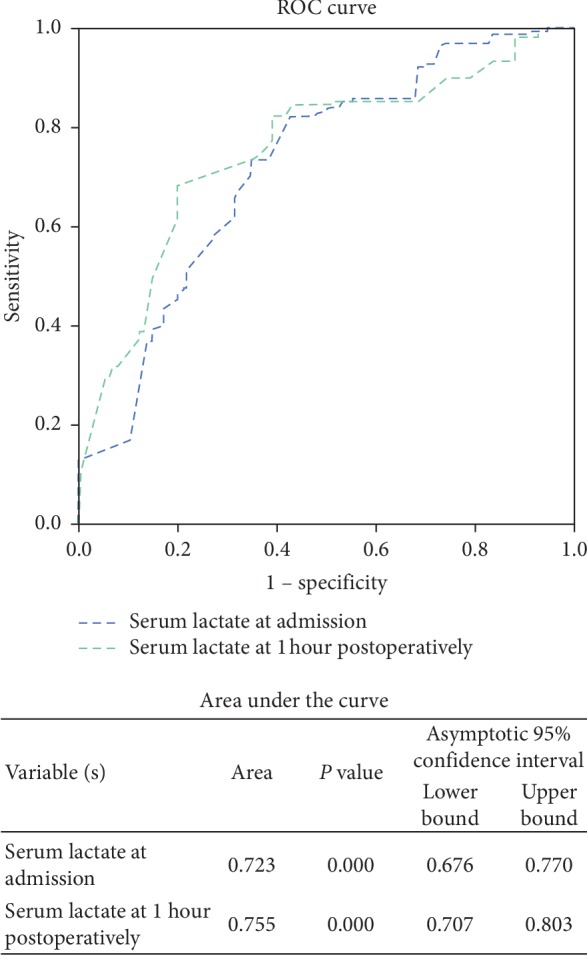
ROC curve and AUC of serum lactate in diagnosing postoperative delirium.

In univariate analysis of perioperative serum lactate levels and lactate clearance as predictors of POD, the OR of serum lactate level on admission was 4.19 (*P* < 0.01) and that 1 h after surgery was 3.83 (*P* < 0.01); however, the OR for change of lactate level was 0.99 (*P*=0.09), the those of ICU admission, ISS, and sex were 4.21 (*P* < 0.01), 2.44, (*P* < 0.01), and 1.9 (*P*=0.02), respectively. In multivariable analysis of predictors of POD, the OR for serum lactate level at 1 h after surgery was 2.83 (*P*=0.01), that for ICU admission was 3.01 (*P*=0.01), and that for ISS was 1.47 (*P* < 0.01). However, serum lactate level on admission, change of lactate level, age, and sex were not significant (Table 4). The area under the curve (AUC) for the receiver operator characteristic (ROC) curve for diagnosing postoperative delirium at admission and that at 1 hour after surgery were 0.72 and 0.76 in our study (Figure 3).

## 4. Discussion

The results of this study confirm the hypothesis that elevated serum lactate levels in elderly trauma patients might be associated with the incidence of POD. Elderly trauma patients with delirium showed significantly higher levels of serum lactate both on admission and 1 h after surgery than patients without delirium. The incidence of POD had a significant relationship with serum lactate levels both on admission and 1 h after surgery. Among elderly trauma patients with POD, both serum lactate levels on admission and 1 h after surgery were significantly higher in patients with major trauma (ISS > 15) than those in patients with minor trauma (ISS ≤ 15). In univariate analysis, serum lactate levels on admission and 1 h after surgery were independently associated (with a 4.2- and 3.8-fold increased odds) with POD in elderly trauma patients. However, in multivariable analysis, only serum lactate level 1 h after surgery was independently associated (with a 2.8-fold increased odds) with POD in elderly trauma patients compared with nondelirium patients.

Elevated serum lactate level has been consistently shown as a prognostic marker of severe injury and mortality in trauma patients, particularly in the elderly. Although these patients may have severely reduced circulation and tissue hypoxia, their blood pressure may be in the normal range because of peripheral vasoconstriction. Thus, elevated serum lactate level in elderly trauma patients may serve as an early indicator of shock before blood pressure or heart rate becomes abnormal [13–15]. Even in trauma patients with stable vital signs, blood lactate level may be useful in differentiating major and minor injuries [14, 15]. As mentioned above, although serum lactate levels both on admission and 1 h after surgery were higher in patients with major trauma than those in patients with minor trauma, the level in both groups was abnormal at >2 mM/L. Exposure to significant longer duration of surgery and anesthesia, a lot of intraoperative fluid transfusion, and higher vasoactive agents used might affect serum lactate level at 1 hour after surgery. The AUC for ROC curve for diagnosing postoperative delirium at admission and that at 1 hour after surgery were 0.72, and 0.76 in our study. A perfect test has an area under the curve of 1; over 0.7 is rated as good or very good for a diagnostic test [16]. Serum lactate level >2 mM/L both on admission and 1 hour after surgery is a good test for postoperative delirium in elderly trauma patients. Serum lactate level at 1 hour after surgery may be better than that on admission as a predicting factor in the elderly trauma patients. The measurement of serum lactate in the present study may facilitate the recognition of occult hypoperfusion, and elevated serum lactate levels both on admission and 1 h after surgery can serve as metabolic indicators of circulatory hemodynamic insufficiency, which is associated with POD [17, 18].

Delirium is common in elderly trauma patients [4, 9, 19]. The present study showed a 38.1% incidence of POD in elderly trauma patients. The comprehensive assessment and early identification of these patients at a risk for POD are important because adequate and timely management could prevent the occurrence of delirium and related adverse outcomes [2, 4–6, 20]. Several theories and prediction models have been suggested to understand the mechanism by which POD develops, although each study had a variable quality and population heterogeneity [4–6, 20]. In the univariate analysis by Norkienė et al. [10], intraoperative and postoperative serum lactate levels were significantly higher in the delirium group than those in the nondelirium group; however, in multivariable analysis, ICU stay and the duration of controlled mechanical ventilation were associated with the development of delirium after cardiac surgery. In the present study, elevated serum lactate levels on admission and 1 h after surgery, ICU admission, and ISS were independent risk factors for the development of POD in multivariable analysis, in contrast with the results of Norkienė et al. [10]. Taking together the results of univariate and multivariable analyses to identify predictors of POD in the present study, elevated serum lactate level 1 h after surgery had a moderate positive correlation with POD and may be suggested as a predictor of POD.

Hypoperfusion in trauma patients cause an adverse immunoinflammatory reaction, which is one of the underlying pathophysiologies of POD [21, 22]. Elderly patients, especially, have reduced physiological reserve, altered pharmacokinetic and pharmacodynamic response, comorbidities, and concomitant use of drugs. Because of these characteristics, elderly patients tend to respond differently from young patients to trauma, surgery, and anesthesia, which could have major effects on the perioperative course [23–25]. The higher ISS, shorter time from admission to surgery, longer duration of surgery and anesthesia, and higher incidence of intraoperative transfusion in the present study may reflect the emergency status and severity of traumatic injury in the studied elderly trauma patients. This study showed that the use of vasoactive drugs and the ISS had a significant association with serum lactate level 1 h after surgery. The higher ISS (major trauma) and use of multiple vasoactive drugs in the delirium group correlated with higher serum lactate levels. The findings of this study may reflect a flow-demand mismatch or loss of appropriate capillary density, such as vasoconstriction or other dysfunctional responses. Although infusion of dopamine decreases or infusion of adrenaline increases serum lactate level in septic patients [26], patients with delirium in the present study showed greater use of the combination of dopamine with adrenaline and had higher serum lactate levels than patients without delirium. As mentioned above, the current patient population was at a higher risk of developing delirium than patients in other studies on factors associated with POD.

Blood lactate level, a parameter often available at the point of care on blood gas analyzers, is useful for assessment of the balance between oxygen demand and availability. The arterial lactate in many studies was demonstrated to be a biomarker for mortality in critically ill trauma patients [1]. Elevated arterial and venous lactate levels are associated with increased mortality; additionally, some studies have shown strong correlation between these two types of lactate values [27, 28]. Arterial or venous serum lactate level in trauma patients should be measured, because it is an easily obtainable biomarker of physiologic derangements in these patients that might also be used to notify the clinician of a potential increased risk of delirium.

The preexisting or nonmodifiable risk factors for delirium at the time of hospital admission include advanced age (>65 years), male sex, alcohol abuse, brain trauma, dementia, hypertension, polypharmacy, and multiple medical comorbidities [2, 3, 29–32]. In the present study, ASA classification, delirium history, polypharmacy, hypertension history, alcohol abuse history, blood transfusion, and use of vasoactive agents were significantly associated with POD. There is sufficient evidence indicating that these variables contribute to the development of POD. The association between sex and delirium has been reported in various clinical settings, including surgery; however, some studies failed to find this association [32, 33]. There was no significant difference in the sex in terms of POD development. The findings of this study are consistent with those of previous studies mentioned above.

The present study has several limitations. First, besides the risk factors for POD evaluated in this study, there might be unidentified or inconsistently reported risk factors that were not included because the etiology of delirium is multifactorial and has not been completely clarified. Second, the type of surgery under anesthesia, operating room capacity, and ICU availability during the time from admission to the initiation of surgery varied in the present study. The change of lactate level may not actually fit with the concept of early detection of hypoperfusion in this study. Therefore, the association of change of lactate level with POD might be weak. Third, we did not perform ROC curve analysis, which might be difficult to analyze diagnostic decision making regarding the association of lactate and POD.

Finally, because mild traumatic brain injury is difficult to diagnose and not readily detectable with brain CT, patients could not be excluded on the basis of a known brain injury. The criteria for identifying a potential mild traumatic brain injury included at least 2 of the following: loss of consciousness for >30 min at the scene, amnesia at or near the time of the event, and a score of 13-14 on the Glasgow Coma Scale on admission [34]. In the present study, as the time of loss of consciousness at the scene was difficult to determine from the medical records, we excluded patients with a documented brain injury.

In conclusion, serum lactate levels both on admission and 1 h after surgery were higher in elderly trauma patients with delirium than those in patients without delirium. In univariate and multivariable analyses, elevated serum lactate level 1 h after surgery was an independent risk factor of POD. Taking the results together, serum lactate level 1 h after surgery may be used as a prediction model of POD development.

## Figures and Tables

**Table 1:** Demographic and perioperative data.

	Delirium group (*n* = 170)	Nondelirium group (*n* = 276)	*P* value
Age (y)	72.9 ± 6.0	73.1 ± 6.4	0.25
Sex (M/F)	96/74	139/137	0.16
Weight (kg)	58.5 ± 7.9	59.3 ± 6.4	0.66
ASA (I/II/III/IV)	8/89/73	42/162/72	<0.01
Alcohol abuse history	44 (25.9)	41 (14.9)	<0.01
Delirium history	60 (35.3)	40 (14.5)	<0.01
Hypertension history	150 (88.2)	222 (80.4)	0.03
Polypharmacy (≥5 medications)	79 (46.5)	75 (27.2)	<0.01
Glasgow Coma Scale	13.60 ± 0.8	13.7 ± 0.7	0.67
Mechanism of injury			<0.01
Fall	16 (9.4)	4 (1.4)	
Penetrating trauma	45 (26.5)	4 (12.7)	
Blunting trauma	8 (4.7)	15 (5.4)	
Motor vehicle collision	60 (35.3)	40 (14.5)	
Others	41 (24.1)	182 (65.9)	
Duration of surgery (min)	400.1 ± 154.5	239.1 ± 111.3	<0.01
Duration of anesthesia (min)	429.6 ± 160.5	301.9 ± 111.0	<0.01
PCA volume (mL)	58.6 ± 5.3	57.0 ± 4.7	0.02
Time from admission to surgery (h)	9.9 ± 7.4	18.8 ± 6.2	<0.01
Intraoperative transfusion (units)	2.9 ± 2.0	0.9 ± 1.6	<0.01
Intraoperative fluid amount (mL)	2500 ± 950	1200 ± 450	<0.01
Hemoglobin level 1 h after surgery (g/dL)	10.7 ± 1.4	10.8 ± 1.0	0.25
Serum lactate level on admission (mM/L)	2.43 ± 0.54	1.90 ± 0.59	<0.01
Serum lactate level 1 h after surgery (mM/L)	2.60 ± 0.76	1.93 ± 0.67	<0.01
Change of lactate level (%)	−11.7 ± 36.7	−5.1 ± 43.0	0.09
Vasoactive agents used			
Dopamine	210 (76.1)	44 (35.9)	<0.01
Norepinephrine or epinephrine	66 (23.9)	126 (74.1)	<0.01
ICU admission	74 (43.5)	67 (24.3)	<0.01

Values are expressed as mean ± standard deviation or number (%) of patients. ASA: American Society of Anesthesiologists, F: female, M: male, ICU: intensive care unit, and PCA: patient-controlled analgesia.

**Table 2:** Correlations between postoperative delirium and perioperative serum lactate levels and change of lactate level.

		Serum lactate level on admission	Serum lactate level 1 hour postoperatively	Change of lactate level
POD	Correlation coefficient (*r*)	0.31	0.36	−0.09
	*P* value	<0.01	<0.01	0.02

POD: postoperative delirium.

**Table 3:** Perioperative serum lactate levels according to the severity of ISS in patients with delirium.

	Major trauma (*n* = 280)	Minor trauma (*n* = 166)	*P* value
Postoperative delirium	127 (45.3)	43 (25.9)	*P* < 0.01
Serum lactate level on admission (mM/L)	2.48 ± 0.52	2.25 ± 0.55	*P*=0.12
Serum lactate level 1 hour after surgery (mM/L)	2.64 ± 0.70	2.47 ± 0.92	*P* < 0.01

Values are expressed as mean ± SD or number (%) of patients. ISS: injury severity score. Major trauma is defined as ISS > 15. Minor trauma is defined as ISS ≤ 15).

**Table 4:** Univariate and multivariable analysis of risk factors associated with delirium development after trauma.

	Univariate analysis		Multivariable analysis	
	Odds ratio	*P* value	Odds ratio	*P* value
Serum lactate level on admission	4.19	<0.01	2.40	0.09
Serum lactate level 1 h after surgery	3.83	<0.01	2.83	0.01
Change of lactate level (%)	0.99	0.09	0.58	0.12
ICU admission	4.21	<0.01	3.01	0.01
ISS	2.44	<0.01	1.47	<0.01
Age	1.34	0.09	1.04	0.13
Sex	1.9	0.02	0.60	0.09

ICU: intensive care unit; ISS: injury severity score.

## Data Availability

The data used to support the findings of this study are available from the corresponding author upon request.
